# Syndecan-1 Levels in Females with Active Rheumatoid Arthritis

**DOI:** 10.3390/jcm13144110

**Published:** 2024-07-14

**Authors:** Norma Alejandra Rodriguez-Jimenez, Fabiola Gonzalez-Ponce, Jorge Ivan Gamez-Nava, Melissa Ramirez-Villafaña, Ana Miriam Saldaña-Cruz, Juan Manuel Ponce-Guarneros, Eva Maria Olivas-Flores, Miguel Angel Macías-Islas, Edgar Ricardo Valdivia-Tangarife, Heriberto Jacobo-Cuevas, Luz Gabriela Ramos-Estrada, Sylvia Totsuka-Sutto, Ernesto German Cardona-Muñoz, Laura Gonzalez-Lopez, on behalf of the Group for the Assessment of Prognosis Biomarkers in Autoimmune Disorders

**Affiliations:** 1 Instituto de Terapeutica Experimental y Clínica, Programa de Doctorado en Farmacología, Departamento de Fisiología, Centro Universitario de Ciencias de la Salud, Universidad de Guadalajara, Guadalajara 44340, Jalisco, Mexico; norma.rodriguezj@academicos.udg.mx (N.A.R.-J.); fabiola.gonzalez@academicos.udg.mx (F.G.-P.); ivangamezacademicoudg@gmail.com (J.I.G.-N.); melissa.ramirez@academicos.udg.mx (M.R.-V.); ana.saldanac@academicos.udg.mx (A.M.S.-C.); juan.ponce4091@academicos.udg.mx (J.M.P.-G.); luzgabriela.re@gmail.com (L.G.R.-E.); sylvia.totsuka@academicos.udg.mx (S.T.-S.); cameg1@gmail.com (E.G.C.-M.); 2 Programa de Maestría en Salud Publica, Departamento de Salud Publica, Centro Universitario de Ciencias de la Salud, Universidad de Guadalajara, Guadalajara 44340, Jalisco, Mexico; 3 Unidad Medica Familiar 97, Instituto Mexicano del Seguro Social, Magdalena 46470, Jalisco, Mexico; 4 Departamento de Anestesiología, Hospital de Especialidades, Centro Medico Nacional de Occidente, IMSS, Guadalajara 44340, Jalisco, Mexico; eolivasflores@gmail.com; 5 Departamento de Neurociencias, Centro Universitario de Ciencias de la Salud, Universidad de Guadalajara, Guadalajara 44340, Jalisco, Mexico; miguelangelmacias@hotmail.com (M.A.M.-I.); ricardovaldiviatangarife@outlook.com (E.R.V.-T.); 6 Programa de Postdoctorado en el Departamento de Psicología Básica, Centro Universitario de Ciencias de la Salud, Universidad de Guadalajara, Guadalajara 44340, Jalisco, Mexico; hjacobocuevas@gmail.com

**Keywords:** rheumatoid arthritis, syndecan-1, disease activity

## Abstract

**Background:** The relationship between serum glycoprotein syndecan-1 and disease activity in rheumatoid arthritis (RA) is still unknown. This study aimed to evaluate whether serum syndecan-1 concentrations are associated with moderate/severe disease activity. **Methods:** Study Design: This was a cross-sectional study. Seventy-five adult women with RA were classified into (a) moderate/severe RA based on the disease activity score, using the erythrocyte sedimentation rate (DAS28-ESR ≥ 3.2, *n* = 50), and (b) RA in remission (DAS28-ESR < 2.6, n = 25). Twenty-five healthy women were taken as the reference group. Syndecan-1 levels were determined using enzyme-linked immunosorbent assay (ELISA). High values of serum syndecan-1 levels (≥24 ng/mL) were used to identify the utility values of this biomarker. **Results:** The patients with RA had higher levels of syndecan-1 than the controls (*p* < 0.001). RA patients with active disease had higher syndecan-1 levels than RA patients in remission (57.6 vs. 23.5 ng/mL, respectively; *p* = 0.002). High syndecan-1 concentrations demonstrated the following utility values for identifying disease activity: sensitivity, 84% (95%CI: 71–93); specificity, 52% (95%CI: 31–72); positive predictive value, 78% (95%CI: 70–84); and negative predictive value, 62% (95%CI: 44–77). **Conclusions:** High syndecan-1 levels have good sensitivity and positive predictive value for identifying disease activity; however, their specificity is limited. Future prospective studies are needed to assess whether syndecan-1 levels can predict treatment failure in RA.

## 1. Introduction

Rheumatoid arthritis (RA) is a systemic disorder of the connective tissue distinguished by chronic inflammation of the synovial joints and periarticular tissues, causing bone erosions and articular cartilage damage [1]. This disease is the most common inflammatory and autoimmune disorder seen in rheumatology consultations [2]. The clinical characteristics of RA include symmetrical inflammation of the small and large joints with predilection of the wrists, metacarpophalangeal, and proximal interphalangeal of the hands and frequent joint stiffness, worsening in the mornings, and constitutional symptoms including fatigue [3]. Around half of RA patients have extra-articular manifestations, including rheumatoid nodules, vasculitis, or interstitial pneumopathy among others [4], whereas laboratory findings include the presence of the rheumatoid factor in 61%, antibodies anti-cyclic citrullinated peptide in 57%, and elevation of acute phase reactants such as C-reactive protein (CRP) and the erythrocyte sedimentation rate (ESR) [5]. 

Several drugs are currently available to treat the disease activity of RA. Existing drugs are classified as (a) conventional synthetic disease-modifying antirheumatic drugs (cs-DMARDs) (such as methotrexate, sulfasalazine, leflunomide, hydroxychloroquine, chloroquine, and azathioprine), (b) biological DMARDs (such as anti-TNF agents, IL-1 receptor antagonists, IL-6 receptor antagonists, anti-CD20 monoclonal antibodies, recombinant human fusion protein of human CTLA4, and the Fc domain of human immunoglobulin G1), and (c) Janus kinase inhibitors [6,7].

One of the most interesting pathogenic mechanisms of inflammation in RA is observed in the synovial tissue. Histopathologically, the synovium in patients with RA shows angiogenesis, the proliferation of lining cells, and the infiltration of mononuclear cells, with edema, damage to capillaries, and vascular congestion with the participation of different molecules involved in these processes [8,9].

Likewise, in other tissues like the pannus synovium, some cell membranes are encased in a glycocalyx, a layer of glycans covering their surface. This layer can be altered during the inflammation observed in RA, affecting various cell membrane properties such as cell adhesion and recognition, thereby modifying the synovial micro-environment. The glycocalyx mainly consists of glycosaminoglycans, such as heparan sulfate (HS) and hyaluronic acid (HA), along with core proteins like syndecans and glypicans [10].

Syndecans are a small family of proteoglycans characterized by their attachment of multiple glycosaminoglycans (GAG) chains to specific serine residues within the protein core. There are four members in the syndecan family; of them, syndecan-1 is a protein located mainly on the surface of epithelial and endothelial cells, and abnormalities of it have been implicated in several chronic disorders [11,12]. 

The biological significance of syndecan-1 hinges on its ability to bind numerous ligands through their heparan sulfate chains such as extracellular matrix (ECM) components, cytokines, chemokines (the formation of gradients on the luminal surfaces of endothelial cells), growth factors and their corresponding receptors, regulating cell adhesion, migration, proliferation, and differentiation through independent signaling mechanism. Syndecans play several roles in matrix interactions and in matrix assembly [13]. 

Syndecan-1 molecules enhance the activation of IL-34 receptors in macrophages [14]. Syndecan-1 is overexpressed in the synovial tissue of RA, participating in the migration and maintenance of mononuclear phagocytes [12]. Additionally, Syndecan-1 is expressed by plasma cells and mononuclear cells in the synovium of patients with RA [15].

**Figure 1:**
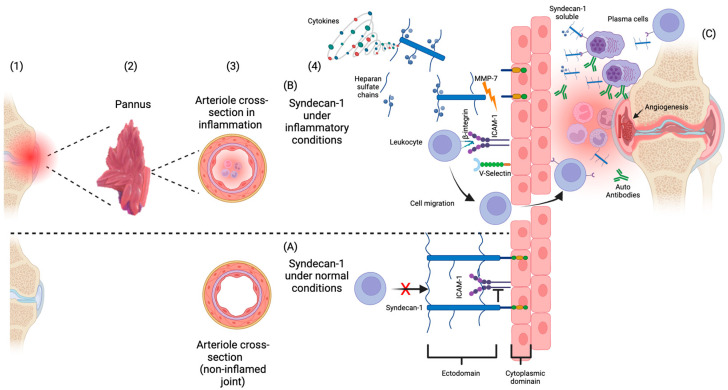
Hypothetical mechanism of Syndecan-1 in joint inflammation in Rheumatoid Arthritis (RA). Sequence: (1) joint in RA with synovial pannus; (2) amplified cross-section of the rheumatoid synovial pannus; (3) cross-section of arteriole in synovial pannus; (4) (**A**) under normal conditions, syndecan-1 decreases the interaction between leukocytes and adhesion molecules in endothelial cells including selectins and ICAM-1, leading to a reduction in the pace leukocytes movement and arresting the leukocyte extravasation. (**B**) Syndecan-1 shedding by metalloproteinases (MMP) and posterior cleavage of heparin sulfate chains by heparanases. Soluble syndecan-1 molecules interplay with extracellular matrix, cytokines, chemokines, and other molecules acting as co-receptors. (**C**) Syndecan-1 in the inflamed joint interacts with receptors in plasma cells, increasing production of autoantibodies and, with receptors of other cells including mononuclear cells, activating inflammation and promoting inflammatory angiogenesis. Created with BioRender.com https://app.biorender.com/illustrations/664b8e4ce2c9c794ee40e3d2 (accessed on 12 June 2024).

In Figure 1A, the syndecan-1 structure and mechanism under normal conditions are shown: the syndecan-1 molecule is linked to endothelial cells avoiding the leukocytes’ joint-to-adhesion-cell molecules (ICAM-1 or V-Selectin) and blocking their rolling to the inflammation site [16]. In Figure 1B, the possible role of syndecan-1 in the chronic inflammation observed in RA is represented. First, the syndecan-1 ectodomains molecule at the cell surface undergoes cleavage by proteolytic enzymes such as metalloproteinases [17]. An increase in the cleavage process produces a rise in the soluble syndecan-1 molecules. Following this increase, more leukocytes interact with adhesion cell molecules, accessing the inflamed joint and thus favoring the persistence of disease activity [18]. Additionally, the binding of soluble syndecan-1 to receptors on the plasma cells increases the production of autoantibodies [19]. Furthermore, soluble syndecan-1 molecules bind the Vascular Endothelial Growth Factor (VEGF) and the Fibroblast Growth Factor (FGF), promoting endothelial cell invasions and leading to angiogenesis, increasing the rheumatoid pannus [20]. 

To date, there is limited information regarding the potential role of syndecan-1 in RA. Deyab et al., in a study of RA patients treated with methotrexate (MTX) alone and patients receiving MTX plus biological agents, identified an association between the C-reactive protein (CRP) and syndecan-1 levels, suggesting a relationship between this molecule and inflammation [21]. In contrast, a study conducted by Kim et al. did not find differences comparing syndecan-1 levels in RA versus controls; however, higher levels of syndecan-1 were observed in SLE compared to the other two groups [22]. Although, in the latter study, there was no information on the clinical characteristics of those in the RA group. Such characteristics could modify syndecan-1 levels, explaining the negative results. 

In RA, the use of antirheumatic drugs could decrease the serum levels of syndecan-1 [21]. 

Consequently, to date, no conclusive information regarding the relationship between syndecan-1 levels and inflammation in RA has been presented. Therefore, this study aimed to evaluate whether syndecan-1 levels are associated with disease activity in RA and to determine the cutoff point of syndecan-1 to identify disease activity in RA patients, as well as to estimate the cutoff values of sensitivity, specificity, positive, and negative predictive values for the diagnosis of disease activity in RA (moderate or severe). 

## 2. Patients and Methods

### 2.1. Design

Cross-Sectional Study.

### 2.2. Study Population

Seventy-five adult females with RA were recruited from a secondary-care hospital (Hospital General Regional #110, Instituto Mexicano del Seguro Social (IMSS) in Guadalajara, Mexico). This study was performed by the Group for the Assessment of Prognosis Biomarkers in Autoimmune Disorders. 

Inclusion and exclusion criteria: We included women aged ≥18 years old, who had RA diagnosed by a rheumatologist, meeting the 1987 criteria for RA [23]. These patients also met the 2010 American College of Rheumatology (ACR) criteria [1]. We excluded patients under treatment with immunosuppressive drugs (cyclophosphamide, mophethyl mycophenolate) or prednisone at >10 mg/day; patients who had received a transfusion in the last 3 months; patients with a serum creatine level of >1.5 mg/dL, alanine aminotransferase (ALT) and aspartate aminotransferase (AST) concentrations >2-fold higher than the reference laboratory values, cancer, thyroid disease, or active infection; and patients who were pregnant or lactating. A control group of clinically healthy women of a similar age range was included to compare the serum syndecan-1 levels. 

### 2.3. Clinical Evaluation

The weight, height, and body mass index (BMI) of the RA patients and the controls were calculated using Quetelet’s formula [24]. In the RA patients, we identified the level of disease activity using the validated Disease Activity Score for 28 joints (DAS28). This is a well-validated index for disease activity in RA, which assesses four parameters: (a) the count of 28 joints for swelling, (b) the count of 28 joints for tenderness, (c) a patient global assessment of disease severity (based on a visual analog scale ranging from 0 to 100 mm), and (d) the results of the erythrocyte sedimentation rate (ESR) or CRP [25]. We computed the DAS28 index using the ESR. According to the score obtained in DAS28-ESR, we classified each RA patient into one of the following groups: (a) patients with moderate or severe disease activity, with a DAS28-ESR score of ≥3.2, and (b) patients with RA in remission, with a DAS28-ESR score of ≤2.6 [26]. Additionally, we assessed the patients’ physical disability using the Spanish-validated version of the Health Assessment Questionnaire Disability Index (HAQ-DI) [27]. This index comprises eight categories related to dressing and grooming, arising, eating, walking, hygiene, reach, grip, and common daily activities, and the patient must indicate whether they can perform these activities without any difficulty (0), with some difficulty (1), or with much difficulty (2), or are unable to perform it at all (3). Finally, an average score of the responses is obtained, giving a value between 0 and 3, with a higher score representing a greater level of disability [27]. 

**Figure 2:**
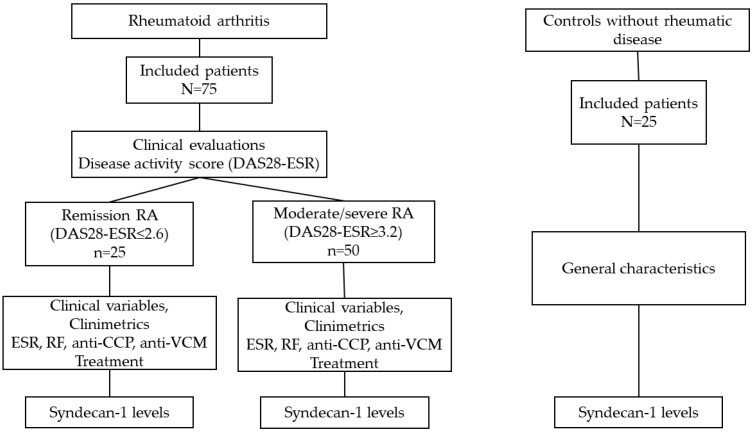
Study flowchart.

We also included a control group of 25 women without rheumatic disease, with similar characteristics to the group of RA women. All patients and controls signed an informed consent form prior to their participation (Figure 2). 

### 2.4. Ethics

The study was approved by the Ethics Committee for Research and Ethics in Health Research of the IMSS (approval code R-2015-1303-16). This research protocol adhered to the tenets outlined in the Declaration of Helsinki given in Fortaleza, Brazil, in 2013. 

### 2.5. Serum Syndecan-1 Determination

After 8 h of fasting, a sample of 5 mL of venous blood was obtained. This sample was centrifuged at 3500 revolutions per minute for 15 min to obtain the serum, which was deposited into coded Eppendorf tubes and stored at −80 °C until processing. The serum syndecan-1 levels were determined using enzyme-linked immunosorbent assay (ELISA) commercial kits (Syndecan-1 (CD138) Human ELISA BioVendor, Brno, Czech Republic ^®^). The characteristics of these kits are a range of determination of 8.0–256 ng/mL, a sensitivity of 4.94 ng/mL, and a coefficient variation of 6.2%.

### 2.6. Statistical Analysis

Quantitative variables were expressed as the median and range, and qualitative characteristics were expressed as frequencies (%). For the comparison of quantitative variables between two groups, a Mann–Whitney U test was performed, and for qualitative variables, a Chi-square test (or Fisher’s exact test, if applicable) was performed. To identify the cut-off value for the syndecan-1 levels in the RA group, we computed high levels of syndecan-1 based on the 75th percentile value of the control group. Therefore, patients were classified as having high levels of syndecan-1 when the levels were ≥24 ng/mL, and normal levels were <24 ng/mL. This cut-off value was used to estimate the sensitivity (95%CI), specificity (95%CI), predictive values (95%CI), and likelihood ratio (LR) of high levels of syndecan-1. In this study, sensitivity was defined as the probability of positive syndecan-1 in patients with active RA disease, and specificity was defined as the probability of negative results for syndecan-1 in RA patients in remission from the disease. The positive predictive value (PPV) was defined as the probability of having active RA disease in the presence of syndecan-1, while the negative predictive value (NPV) was defined as the probability of having RA disease in remission in the presence of a negative result for syndecan-1. We computed 95% confidence intervals (95%CI) for the utility values of syndecan-1. Statistical significance was set at the 0.05 level. The analyses were performed using the statistical software SPPS Statistics Version 24.

## 3. Results

### 3.1. Clinical Characteristics of the Patients Included in the Study

Table 1 describes the clinical characteristics of the patients with RA. The patients included in our study had a median age of 54 years, with a disease duration of 6 (0.58–60) years. The median of the DAS28-ESR score was 5.81 (0.9–7.3). High levels of syndecan-1 (≥24 ng/mL) were present in 72% of patients with RA. Other characteristics, such as the HAQDI score, CRP, ESR, RF, anti-CCP, anti-MCV, and the frequency of use of each DMARD, are described in Table 1.

### 3.2. Comparison of the Characteristics between the RA Patients and the Controls

As shown in Table 2, we compared the general characteristics between the RA patients and the controls. Patients’ age, weight, and BMI were similar between both groups, whereas the syndecan-1 levels were higher in the RA group than in the controls (median: 48.5 vs. 13.4, respectively; *p* < 0.001) (Table 2).

### 3.3. Comparison of Syndecan-1 Serum Levels between the Control Group, Remission RA, and Moderate or Severe Disease Activity in RA (Active RA)

**Figure 3:**
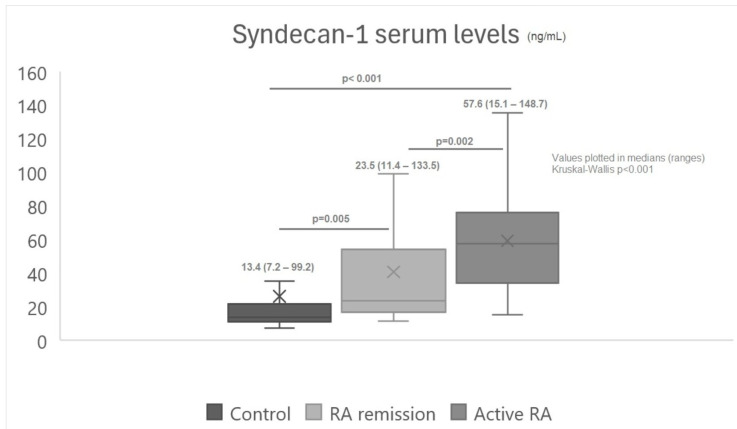
Comparison of syndecan-1 serum levels between the control group, remission RA disease, and active RA disease. The Kruskal–Wallis test for independent samples was performed. The Mann–Whitney U test was performed to identify differences between each group. The results were between control and inactive RA (*p* = 0.005), between control and active RA (*p* < 0.001), and between inactive RA and active RA (*p* = 0.002).

We compared the syndecan-1 levels among the three groups: the control group, the moderate or severe disease activity RA group (active RA), and the remission RA group (RA remission) (Figure 3). The syndecan-1 levels were higher in the moderate or severe disease activity RA group (active RA) compared with the other two groups (Figure 3). 

### 3.4. Comparison of Clinical Characteristics between Remission/Mild RA vs. Moderate/Severe RA

Using the data presented in Table 3, we compared RA patients with active disease (group A) to RA patients in remission (group B). In patients with active disease, we observed higher syndecan-1 levels than in patients in remission (median of syndecan-1 levels: 57.6 vs. 23.5, respectively; *p* = 0.002). Considering the cutoff of ≥24 ng/mL for a high level of syndecan-1, in RA patients with active disease, 84% had levels higher than the cutoff, compared with 48% of the patients in remission (*p* = 0.001). Other characteristics observed in RA with active disease were a high HAQ-DI and higher ESR (Table 3).

### 3.5. Utility Values of Syndecan-1 Levels ≥24 ng/mL, for Biomarker of Active Rheumatoid Arthritis

Table 4 shows the utility values of high syndecan-1 levels (≥24 ng/mL) used to identify disease activity in RA. We observed the following utility values for identifying disease activity: sensitivity, 84% (95%CI: 71–93), specificity, 52% (95%CI: 31–72), positive predictive value, 78% (95%CI: 70–84), negative predictive value, 62% (95%CI: 44–77), positive likelihood ratio (LR+) 1.75 (1.14–68), and negative likelihood ratio (LH−) 0.31 (0.15–0.64) (Table 4).

## 4. Discussion

We observed higher syndecan-1 serum levels in the RA patients compared to the controls. The syndecan-1 levels were also higher in RA patients with active disease than in those with inactive RA, according to the DAS28-ESR. When we analyzed high syndecan-1 levels according to the cutoff value of ≥24 ng/mL, higher syndecan-1 levels had high sensitivity (84%) for identifying disease activity in RA; although, this cutoff value had low specificity (52%). The positive predictive value of syndecan-1 levels with this cutoff for identifying disease activity in patients with higher levels of syndecan-1 was 78%.

### 4.1. Syndecan-1 Levels in RA: Comparisons with Other Studies

We observed higher syndecan-1 serum levels in RA patients compared to the controls. These results differed from those observed by Kim et al. in a sample of Korean patients with RA, SLE, and controls [22]. Those authors observed higher values of syndecan-1 in SLE patients compared with the two other groups, although they did not identify differences in Syndecan-1 levels between RA patients and controls [22]. Nevertheless, the lack of a description of clinical characteristics in RA patients included in that study makes it difficult to interpret their results.

In our study, the syndecan-1 levels were higher in RA patients with active disease than in those with inactive RA, according to the DAS28-ESR. In a study of Norwegian patients, Deyab et al. found that syndecan-1 levels were associated with CRP levels, indicating an interrelation between syndecan-1 and inflammation [21]. 

### 4.2. Cutoff Value of High Syndecan-1 Levels and Utility Values for Identifying Disease Activity

This study used a cutoff value of ≥24 ng/mL to determine high syndecan-1 levels in our patients, and this was found to be helpful for identifying patients with moderate and severe disease activity. Using this cutoff value, high syndecan-1 levels had good sensitivity for identifying patients with active disease (84%), although the specificity was low (52%). These findings suggested that the levels of these molecules are linked to some processes of disease activity in RA that deserve further investigation.

### 4.3. Evidence in the Literature of the Role of Syndecan-1 in Inflammatory Processes

Syndecan-1 is a transmembrane proteoglycan found in the apical and basolateral part of endothelial cells [28,29]. The ectodomains of this molecule can interact with cytokines, proteases, and growth factors due to their long chains of glycosaminoglycans, allowing them to maintain cellular homeostasis under normal and pathological conditions, such as inflammatory processes, from the recruitment of leukocytes until the resolution of inflammation [30]. Syndecan-1 is eliminated by metalloproteinases that cleave the ectodomains of the core protein and then release syndecan-1 from the endothelium into circulation, resulting in an increase in serum levels [31,32]. 

One of the main functions of syndecan-1 is the regulation of leukocyte migration and cytokine expression [12]. These leukocytes pass from the bloodstream to adhere to sites of inflammation and nearby sites of the inflammatory process [33]. 

Syndecan-1 is found in either membrane-bound or soluble ectodomain forms following proteolytic cleavage. A category of membrane-bound enzymes known as sheddases, including matrix metalloproteinase-2 (MMP-2), MMP-7, MMP-9, and a disintegrin and metalloproteinase domain-containing protein-17 (ADAM-17), cleave Sindecan-1 at the juxtamembrane site, generating soluble ectodomains. This process typically accelerates under inflammatory diseases and other conditions [28,30,34]. Additionally, the elevation of soluble syndecan-1 increases the recruitment of leukocytes and chemokines [16]. Therefore, these mechanisms can explain the association between the high levels of syndecan-1 and inflammation in RA patients.

Due to the evidence of the elevation of syndecan-1 in its soluble form, it has been proposed that it could be a good biomarker of inflammation [16]. We suggest that in future studies, it would be interesting to analyze the serum levels of syndecan-1 together with interleukins such as TNF-α, IL-17, or IL-8, among other markers of inflammation, for the purpose of differentiating RA in the early stages from long-lasting RA.

### 4.4. Treatment and Syndecan-1 Levels

The use of glucocorticoids in the treatment of RA is common [35]. In our study, the frequency of use was 77%, and the group that presented the highest proportion of glucocorticoid use was the moderate/severe RA group (86% vs. 28%, *p*-value 0.032). Glucocorticoids influence proteoglycans, although the exact effect has not been completely elucidated. The tendency for glucocorticoids to decrease the number of proteoglycans in animal cartilage has been reported; however, high doses of glucocorticoids were used (80 mg/mL × 20 weeks) [36]. In our study, all patients were receiving a prednisone dose of ≤10 mg/day. 

Deyab et al. found that treatment with antirheumatic drugs decreased the serum levels of syndecan-1. In our study, there was no difference in the use of synthetic DMARDs between the RA in remission vs. RA with moderate/severe disease activity groups [21].

### 4.5. Strengths of the Present Study

Our study identified the differences in the serum levels of syndecan-1 between RA patients with active disease and those in remission. It was also the first study to assess the sensitivity and specificity of high syndecan-1 levels to identify active RA. According to our results, high syndecan-1 levels have good sensitivity (84%) but limited specificity (52%) for identifying moderate/severe disease activity in RA. Therefore, we suggest that syndecan-1 levels should be evaluated in RA patients with moderate or severe disease activity in further studies to identify whether the levels of this molecule could be predictive of different outcomes in RA patients. 

### 4.6. Limitations of the Present Study

This study had limitations that should be addressed in future studies. The first were those associated with cross-sectional studies: we only obtained a snapshot of a single point in time of the relationship between the levels of syndecan-1 and the severity of disease activity, but whether these levels were present prior to inflammation could not be tested using this design. Another limitation was that cross-sectional studies do not allow the analysis of modifications in the levels of syndecan-1 concentrations or whether other factors, such as the use of pharmacotherapy to treat the disease (disease-modifying antirheumatic drugs, biological agents, or glucocorticoids) can modify the levels and activity of the molecule. Therefore, studies that control the type of therapeutics used in longitudinal designs are required to explore the relationship between the therapeutic response and changes in the levels of syndecan-1. Finally, this study does not represent the entire population of RA patients since only women were included. Future research will involve RA patients of both sexes. 

## 5. Conclusions

In conclusion, elevated syndecan-1 levels are related to disease activity. High syndecan-1 levels have good sensitivity and a moderate positive predictive value for identifying disease activity; however, the specificity of this approach is limited. Future follow-up studies are necessary to identify whether the elevated syndecan-1 levels, as defined by our study, can predict treatment failure in controlling the disease severity in RA patients, as well as to evaluate whether syndecan-1 can be used as a therapeutic target to control disease activity.

## Figures and Tables

**Table 1:** Characteristics of patients with rheumatoid arthritis included in the study.

	RA Patients n = 75
Age, median (range)	54 (23–93)
BMI (kg/m^2^) median (range)	27.9 (15–43.8)
Disease duration, median (range)	6 (0.58–60)
HAQ-DI, median (range)	0.78 (0–2.56)
DAS28 index, median (range)	5.81 (0.9–7.3)
**DAS28 ≤ 2.6, frequency (%)**	25 (33)
**DAS28 ≥ 3.2, frequency (%)**	50 (67)
CRP (mg/dL), median (range)	9.1 (0.8–163)
ESR (mm/h), median (range)	27 (1–64)
Rheumatoid factor (UI/mL), median (range)	29.9 (1–2188)
Anti-CCP (U/mL), median (range)	18.5 (0.01–705)
Anti-MCV (U/mL), median (range)	110 (4.4–1050)
Syndecan-1 (ng/mL), median (range)	48.5 (11.42–148.69)
High levels Syndecan-1 (p75) ≥ 24 ng/mL	54 (72)
Glucocorticoids, frequency (%)	58 (77)
Methotrexate, frequency (%)	54 (72)
Monotherapy, frequency (%)	31 (42)
Combined therapy, frequency (%)	23 (30)
Leflunomide, frequency (%)	26 (35)
Monotherapy, frequency (%)	16 (21)
Combined therapy, frequency (%)	10 (14)
Sulfasalazine, frequency (%)	16 (21)
Monotherapy, frequency (%)	1 (1)
Combined therapy, frequency (%)	15 (20)
Chloroquine, frequency (%)	7 (9)
Monotherapy, frequency (%)	1 (1)
Combined therapy, frequency (%)	6 (8)
Azathioprine, frequency (%)	4 (5)
Combined therapy, frequency (%)	4 (5)
Biologic DMARDs, frequency (%)	2 (3)
Etanercept, frequency (%)	2 (3)

Quantitative variables expressed in medians and ranges; qualitative variables expressed in frequency and (%). Abbreviations: RA: rheumatoid arthritis; BMI: Body mass index; DMARDs: disease-modifying anti-rheumatic drugs; HAQ-DI: Health Assessment Questionnaire Disability Index; DAS: Disease Activity Score; CRP: C-Reactive Protein; ESR: erythrocyte sedimentation rate; Anti-CCP: Anti-Cyclic Citrullinated Peptide; Anti-MCV: Anti-Mutated Citrullinated Vimentin.

**Table 2:** Comparison between selected characteristics of patients with RA vs. controls.

	RA n = 75	Controls n = 25	*p*-Value
Age, median (range)	54 (23–93)	56 (22–87)	0.902
Weight, (kg) median (range)	66 (36–116)	67 (42–104)	0.528
BMI (kg/m^2^), median (range)	27.9 (15–43.8)	27.7 (16.8–38.7)	0.598
Syndecan-1 (ng/mL)	48.5 (11.4–148.7)	13.4 (7.2–99.2)	**<0.001**

Quantitative variables expressed in medians and ranges and compared by Mann–Whitney U tests; Abbreviation: RA: rheumatoid arthritis, BMI: Body mass index.

**Table 3:** Comparison of clinical characteristics between remission RA vs. moderate/severe RA.

	Moderate/Severe RA n = 50	Remission RA n = 25	*p*-Value
Age, median (range)	51.5 (23–93)	61 (33–77)	0.082
BMI (kg/m^2^) median (range)	27.9 (15–43.8)	27.9 (20.8–38.1)	0.799
Disease duration, median (range)	6 (0.58–60)	8 (2–30)	0.407
HAQ-DI, median (range)	1.05 (0–2.56)	0.11 (0–1.11)	**<0.001**
ESR (mm/h), median (range)	32 (10–64)	18 (1–39)	**<0.001**
Rheumatoid factor (UI/mL), median (range)	30 (1–2187)	29 (5–319)	0.649
Anti-CCP (U/mL), median (range)	11.9 (0.01–704.9)	1.96 (0.67–242.8)	0.614
Anti-MCV (U/mL), median (range)	133.5 (4.4–1050.0)	5.73 (3.0–1816.7)	0.214
Syndecan-1 (ng/mL), median (range)	57.6 (15.1–148.7)	23.5 (11.4–133.5)	**0.002**
High levels Syndecan-1 (p75) ≥24 ng/mL	42 (84)	12 (48)	**0.001**
Glucocorticoids, frequency (%)	42 (86)	16 (28)	**0.032**
Synthetic DMARDs, frequency (%)	50 (100)	25 (100)	1.000
Biologic DMARDs, frequency (%) *	2 (4)	0 (0)	0.550 *

Quantitative variables expressed in medians and ranges and compared by Mann–Whitney U Test; qualitative variables expressed in frequency and (%) and compared by Chi-square tests or exact Fisher tests (*). Abbreviations: RA: rheumatoid arthritis; BMI: Body mass index; DMARDs: disease-modifying anti-rheumatic drugs; HAQ-DI: Health Assessment Questionnaire Disability Index; ESR: erythrocyte sedimentation rate; Anti-CCP: Anti-Cyclic Citrullinated Peptide; Anti-MCV: Anti-Mutated Citrullinated Vimentin.

**Table 4:** Utility values of syndecan-1 levels ≥ 24 ng/mL, as biomarker of moderate or severe disease activity in rheumatoid arthritis (active RA).

Utility Values of the Assay	Active RA
Sensitivity % (95% CI)	84 (71–93)
Specificity % (95% CI)	52 (31–72)
Positive predictive value % (95% CI)	78 (70–84)
Negative predictive value % (95% CI)	62 (44–77)
LR+	1.75 (1.14–2.68)
LR−	0.31 (0.15–0.64)
Prevalence	67 (55–77)

LR+: positive likelihood ratio; LR−: negative likelihood ratio.

## Data Availability

The data used to support the findings of this study are available upon request to the author for correspondence: Laura Gonzalez-Lopez:lauraacademicoudg@gmail.com.
